# Pathogenic germline variants in *BRCA1* and *TP53* increase lung cancer risk in Chinese

**DOI:** 10.1002/cam4.6692

**Published:** 2023-11-06

**Authors:** Bing Wei, Jiadong Zhao, Jun Li, Junnan Feng, Manman Sun, Zhizhong Wang, Chao Shi, Ke Yang, Yue Qin, Jing Zhang, Jie Ma, Hui Dong

**Affiliations:** ^1^ Department of Molecular Pathology, Henan Key Laboratory of Molecular Pathology The Affiliated Cancer Hospital of Zhengzhou University & Henan Cancer Hospital Zhengzhou Henan China; ^2^ Nanjing Shenyou Institute of Genome Research Nanjing Jiangsu China; ^3^ Department of Gastroenterology, Shanghai Key Laboratory of Pancreatic Diseases Shanghai General Hospital, Shanghai Jiao Tong University School of Medicine Shanghai China

**Keywords:** *BRCA1*, Chinese, germline variants, lung cancer, *TP53*

## Abstract

**Backgroud:**

Multiple studies have identified pathogenic germline variants in cancer susceptibility genes (CSGs) in Chinese lung cancer patients; however, accurate assessment of these variants' contributions to cancer predisposition is always hampered by the absence of data on the prevalence of these variants in the general population. It is necessary to conduct a large‐scale case–control study to identify CSGs that significantly increase the risk of lung cancer.

**Materials and methods:**

We performed targeted sequencing of a CSGs panel in 1117 lung cancer patients and 16,327 controls from the general Chinese population.

**Results:**

In comparison to controls, lung cancer patients had a considerably higher prevalence of pathogenic and likely pathogenic (P/LP) variations. Among lung cancer patients, 72% of P/LP variants carriers did not have a family cancer history, who would be ignored if germline testing was only provided for patients meeting family history‐based criteria. Furthermore, compared to individuals with late‐onset lung cancer, patients with early‐onset lung cancer had a considerably higher prevalence of P/LP variations. With odds ratios (ORs) ranging from 4‐fold (*BRCA1*: OR, 4.193; 95%CI, 1.382–10.768) to 29‐fold (*TP53*: OR, 29.281; 95%CI, 1.523–1705.506), P/LP variants in the *BRCA1* and *TP53* genes were discovered to be strongly related to increased lung cancer risk. Additionally, with ORs ranging from 7.322‐fold to infinity, we discovered 23 variations previously categorized as non‐P/LP variants were highly enriched in lung cancer patients.

**Conclusion:**

Our findings indicated that P/LP variants in *BRCA1* and *TP53* conferred increased risk of lung cancer in Chinese.

## 1 INTRODUCTION

Lung cancer is the second most common cancer worldwide, with an estimated 2.2 million new cancer cases in 2020.^1^ It is well known that various environmental factors, such as smoking, exposure to ambient air pollution or smoky coal, and occupational exposure to asbestos, contribute to the incidence of lung cancer.^2^, ^3^, ^4^ Additionally, substantial evidence showed that genetic factors were also a significant contributor to the risk of lung cancer. Through a large prospective twin‐based investigation, it was estimated that the overall heritability of lung cancer was 18%.^5^ Previous studies identified multiple risk loci of lung cancer through genome‐wide association studies (GWAS), while GWAS‐derived polygenic risk score (PRS) was proved to be an effective tool to predict lung cancer risk.^6^, ^7^ Notably, the incidence of lung cancer among light smokers with high PRS was higher than that among heavy smokers with low PRS, indicating that genetic factors were also important lung cancer predictors and might be used in population screening programs.^7^ These germline‐derived genetic biomarkers may be especially helpful in screening because they do not alter throughout a person's life despite changes in other risk variables.

In addition to GWAS‐identified risk loci, rare germline variants of the *EGFR*, *PARK2*, and *YAP1* genes have been reported to confer a high risk of familial lung cancer, while pathogenic germline variants of the *BRCA1*, *BRCA2*, *TP53*, and *ERBB2* have been found to be enriched in sporadic lung cancer patients.^8^, ^9^, ^10^, ^11^, ^12^, ^13^ Hu et al^11^ demonstrated that there were 64 (1.03%) carriers of *BRCA1* and *BRCA2* pathogenic germline variants in 6220 Chinese non‐small cell lung cancer (NSCLC) patients, with *BRCA2* variants being the most predominant. Similarly, pathogenic germline variants in *BRCA1* and *BRCA2* were detected in 6 (0.34%) and 16 (0.79%) participants, respectively, in another study which involved 1764 Chinese lung cancer patients.^13^ When expanding to multiple cancer susceptibility genes (CSGs) panel, the prevalence of pathogenic germline variants in Chinese lung cancer patients increased to 3.8%.^13^, ^14^ In the TCGA cohort, 5.5% of 1087 lung cancer patients were found to carry pathogenic germline variants using a 152‐gene panel.^15^ It was suggested that multigene panel testing would improve the discovery of heritable variants over targeted testing based on guidelines.^16^

Identification of a genetic predisposition would substantially assist the carrier in selecting risk‐reducing interventions before developing cancer, as well as in making appropriate treatment options once diagnosed with cancer. Previous investigations on the role of CSGs germline variants in lung cancer were mainly focused on patients, while information on the prevalence of germline variants in the general population was always absent which impeded the precise evaluation of their contributions to cancer risk. In this study, we aimed to investigate the risk of CSGs germline variants to lung cancer by assessing their prevalence in both unselected lung cancer patients and the general population in China.

## 2 PARTICIPANTS AND METHODS

### 2.1 Participants

Patients who had a current or previous diagnosis of lung cancer were recruited from Henan Cancer Hospital. Patients were unselected for histopathology, stage of disease, and family history of cancer. Individuals from the general population who underwent routine health checkup and denied personal cancer history were recruited as controls. Blood or saliva samples were collected from participants to extract genomic DNA after anonymization. Clinical information including sex, age, histopathology, smoking, and family cancer history were collected from lung cancer patients, while only sex and age were available from controls. The study was performed in accordance with the Declaration of Helsinki and approved by the Ethics Committee of Henan Cancer Hospital and Shanghai Ethics Committee for Clinical Research. Written informed consent was obtained from all subjects.

### 2.2 Targeted sequencing of CSGs

Genomic DNA was extracted from whole blood or saliva using MagMAX™ DNA Multi‐Sample Ultra 2.0 Kit (Thermo Fisher Scientific, Waltham, MA, USA). An amplification‐based panel designed by Fluidigm D3 Assay Design program (Fluidigm, San Francisco, CA, USA) was applied to amplify exons and exon/intron boundaries of *APC*, *ATM*, *BRCA1*, *BRCA2*, *CDH1*, *CHEK2*, *MLH1*, *MSH2*, *MSH6*, *PALB2*, *PTEN*, *STK11*, and *TP53* genes. PCR amplification of targeted sequences were performed using targeted DNA Seq Library Preparation reagents on Fluidigm LP 192.24 chip (Fluidigm). PCR products were collected and then purified using Agencourt AMPure XP (Beckman Coulter, Brea, CA, USA) before being sequenced on HiSeq X Ten instrument (Illumina, San Diego, CA, USA). The sequencing coverage and quality statistics for each sample are summarized in Table S1.

### 2.3 Germline variant calling and classification

Germline variant calling was carried out as described previously.^17^ Briefly, sequencing reads were mapped to human reference genome (hg19) and germline variants were called using GATK. NM_000038.5 (*APC*), NM_000051.3 (*ATM*), NM_007294.3 (*BRCA1*), NM_000059.3 (*BRCA2*), NM_004360.5 (*CDH1*), NM_007194.4 (*CHEK2*), NM_000249.3 (*MLH1*), NM_000251.2 (*MSH2*), NM_000179.2 (*MSH6*), NM_024675.3 (*PALB2*), NM_000314.7 (*PTEN*), NM_000455.4 (*STK11*), and NM_000546.5 (*TP53*) were used as reference sequences for variant calling. Variants with sequencing depth ≥ 50× and altered allele frequency ≥ 20% were retained for further annotation. Variants were annotated using ANNOVAR, and submitted to searching genome databases, including ClinVar, dbSNP150, ExAC, 1000 Genomes Project, and gnomAD. According to the American College of Medical Genetics and Genomics‐Association for Molecular Pathology (ACMG‐AMP) guidelines for interpretation of variants,^18^ variants were classified as pathogenic (P), likely pathogenic (LP), variant of uncertain significance (VUS), likely benign (LB), and benign (B). All variants classified as P/LP were further validated by Sanger sequencing.

### 2.4 Statistics analysis

Comparisons between different groups were performed using Mann–Whitney test, chi‐square test, or Fisher exact test as appropriate. *p* value was calculated using a 2‐sided hypothesis test, and *p* < 0.05 was considered statistically significant. The Benjamini‐Hochberg method was applied for multiple hypothesis testing. All statistical analysis was performed using R package (version 3.5.3).

## 3 RESULTS

### 3.1 Cohort characteristics

A total of 1117 unselected lung cancer patients including 691 males and 426 females were enrolled in the study. The characteristics of patients including sex, age, histopathology, and smoking status are shown in Table 1. In addition, 16,327 controls from the general population who denied personal cancer history were also involved in the study. The distribution of sex and age of controls are listed in Table 1. The mean age of the controls (39.7 ± 11.3 for males and 38.1 ± 8.8 for females) was significantly younger than that of patients (59.8 ± 9.79 for males and 58.1 ± 10.9 for females) (*p* < 0.01). Notably, 15,362 (94.1%) of the controls were females, indicating that females were more likely to undergo health checkup and genetic testing, which was consistent with previous investigations.^19^, ^20^, ^21^, ^22^ Among the 1117 patients, 704 (63%) had adenocarcinoma lung cancer, 198 (17.8%) had squamous cell lung cancer, 199 (17.8%) had small cell lung cancer, and 16 (1.4%) had lung cancer of other histopathological categories. Squamous cell lung cancer was more common in males (26.9%) than in females (2.8%), while adenocarcinoma lung cancer was more common in females (83.3%) than in males (50.5%) (*p* < 0.01). The majority of male patients (72.6%) self‐reported being smokers, while only two female patients (0.5%) were smokers. A total of 218 patients (19.5%) self‐reported having at least one first‐degree relative with cancer. Detailed information on family cancer history is provided in Table S2.

**TABLE 1:** Characteristics of lung cancer patients and controls.

	Lung cancer patients (*n* = 1117)			Controls (*n* = 16,327)		
	Male (*n* = 691)	Female (*n* = 426)	Subtotal	Male (*n* = 965)	Female (*n* = 15,362)	Subtotal
Age						
Mean ± SD	59.8 ± 9.79	58.1 ± 10.9	/	39.7 ± 11.3	38.1 ± 8.8	/
Median	61	57	/	38	36	/
Range	30–83	30–88	/	19–75	19–82	/
Histopathology						
Adenocarcinoma	349 (50.5%)	355 (83.3%)	704 (63%)	/	/	/
Squamous cell	186 (26.9%)	12 (2.8%)	198 (17.8%)	/	/	/
Small cell	144 (20.8%)	55 (12.9%)	199 (17.8%)	/	/	/
Other	12 (1.7%)	4 (1%)	16 (1.4%)	/	/	/
Smoking status						
Yes or ever	502 (72.6%)	2 (0.5%)	504 (45.1%)	/	/	/
Never	65 (9.4%)	414 (97.2%)	479 (42.9%)	/	/	/
Not available	124 (18.0%)	10 (2.3%)	134 (12.0%)	/	/	/

### 3.2 Germline variants in 1117 lung cancer patients

A multigene panel was applied in targeted sequencing, and germline variants were identified in both lung cancer patients and controls. We identified 1257 unique variants in 1117 lung cancer patients, with pathogenic and likely pathogenic variants (P/LP) accounting for 2.0%, benign and likely benign variants (B/LB) accounting for 30.8%, variants of uncertain significance (VUS) accounting for 14.2%, variants with conflicting interpretations of pathogenicity accounting for 8.2%, and unclassified variants (variants that are not recorded in ClinVar) accounting for 44.8% (Table 2). Integration of epidemiological, genetic, and/or histopathological data, as well as in silico prediction and/or in vitro functional analysis, was suggested to be the best way to interpret the role and mechanism of unclassified variants in cancer risk, which always required collaboration with other groups.^23^ Therefore, in the future, we will collaborate with other research teams to categorize these unclassified variants unambiguously.

**TABLE 2:** Clinical significance of unique variants identified in lung cancer patients and controls.

Clinical significance of variants	Lung cancer patients	Controls
Benign and/or likely benign	388 (30.8%)	1217 (16.2%)
Uncertain significance	178 (14.2%)	1336 (17.8%)
Pathogenic and/or likely pathogenic	25 (2.0%)	150 (2.0%)
Conflicting interpretations of pathogenicity	103 (8.2%)	432 (5.8%)
Unclassified	563 (44.8%)	4370 (58.2%)
Total	1257	7505

**FIGURE 1:**
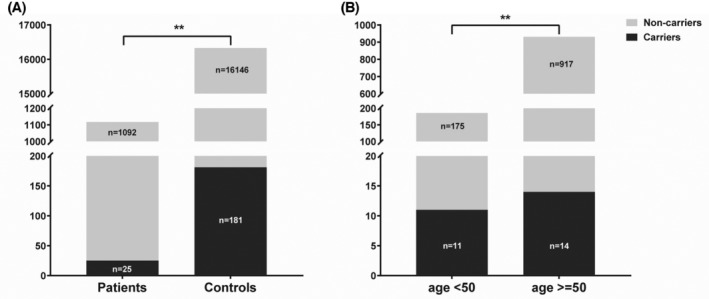
Prevalence of P/LP variants in lung cancer patients and controls. (A) Prevalence of P/LP variants in 1117 lung cancer patients and 16,327 controls. A total of 25 and 181 P/LP variants were identified in patients and controls respectively, and the prevalence of P/LP variants was significantly higher in patients (25/1117, 2.24%) than in controls (181/16327, 1.11%). (B) Prevalence of P/LP variants in early‐onset and late‐onset lung cancer patients. A total of 11 P/LP variants were identified in 186 early‐onset patients, and 14 P/LP variants were identified in 931 late‐onset patients. Compared to late‐onset patients, the prevalence of P/LP variants was significantly higher in early‐onset patients (5.9% vs. 1.5%). ** represents *p* value <0.01.

A total of 15 P/LP variants were found in 691 male patients, while 10 P/LP variants were found in 426 female patients (Table 3 and Table S3). The carrier frequency of P/LP variants was quite similar in male (15 out of 691, 2.2%) and female (10 out of 426, 2.3%) patients. Among the 25 carriers, 15 had adenocarcinoma lung cancer, six had squamous cell lung cancer, three had small cell lung cancer, and one had lung cancer of other histopathological categories. Only seven carriers (28%) self‐reported having a family history of various malignancies including lung cancer, implying that 72% of the carriers would be missed if germline testing was only provided for patients who met family history‐based criteria (Table S3). Notably, the prevalence of P/LP variants was 5.9% in patients with early‐onset lung cancer (age < 50 years old), while it was significantly lower (1.5%) in patients with late‐onset cancer (age ≥ 50 years old), suggesting that P/LP variants might play a more importantly role in younger patients (Figure 1).

**TABLE 3:** Prevalence of P/LP variants in lung cancer patients and controls.

Genes	Lung cancer patients (*n* = 1117)				Controls (*n* = 16,327)				Odds ratio	95% CI	*p* Value	Adjusted *p* Value^b^
	Male (*n* = 691)	Female (*n* = 426)	Subtotal	Prevalence	Male (*n* = 965)	Female (*n* = 15,362)	Subtotal	Prevalence				
*ATM*	1	3	4	4/1117 (0.36%)	3	31	34	34/16327 (0.21%)	1.722	0.44–4.84	0.305	0.366
*BRCA1*	6	0	6	6/1117 (0.54%)	1	20	21	21/16327 (0.13%)	4.193	1.38–10.77	0.006	0.036
*BRCA2*	2	5	7	7/1117 (0.63%)	2	51	53	53/16327 (0.32%)	1.936	0.74–4.29	0.106	0.212
*CHEK2*	2	0	2	2/1117 (0.18%)	0	18	18	18/16327 (0.11%)	1.625	0.18–6.80	0.369	0.369
*PALB2*	3	1	4	4/1117 (0.36%)	3	29	32	32/16327 (0.20%)	1.830	0.47–5.17	0.289	0.366
*TP53*	1	1	2	2/1117 (0.18%)	0	1^a^	1	1/16327 (0.01%)	29.281	1.52–1705.51	0.012	0.036
*APC*	0	0	0	0	0	1	1	1/16327 (0.01%)	/	/	/	
*CDH1*	0	0	0	0	0	2	2	2/16327 (0.01%)	/	/	/	
*MLH1*	0	0	0	0	1	3	4	4/16327 (0.02%)	/	/	/	
*MSH2*	0	0	0	0	0	0	0	0	/	/	/	
*MSH6*	0	0	0	0	1	12	13	13/16327 (0.08%)	/	/	/	
*PTEN*	0	0	0	0	0	1	1	1/16327 (0.01%)	/	/	/	
*STK11*	0	0	0	0	0	1	1	1/16327 (0.01%)	/	/	/	
Subtotal	15	10	25	25/1117 (2.24%)	11	170	181	181/16327 (1.11%)	2.042	1.282–3.129	0.002	/

^a^ The individual carried two P/LP variants (one in *TP53* and one in *PALB2*) simultaneously.

^b^ Benjamini‐Hochberg adjusted *p* value.

### 3.3 Germline variants in 16,327 controls

A total of 7505 unique germline variants were found in 16,327 controls, of which 150 (2%) were P/LP variants, 1217 (16.2%) were B/LB variants, 1336 (17.8%) were VUS, 432 (5.8%) were variants with conflicting interpretations of pathogenicity, and 4370 (58.2%) were unclassified variants (Table 2). The majority of P/LP variants (129 out of 150) were found only once, whereas 21 of them were recurrent in two or three individuals. Notably, one person had two P/LP variants (one in *TP53* and one in *PALB2*) simultaneously (Table 3 and Table S4). Totally, there were 180 carriers carrying 181 P/LP variants, and no difference in the carrier frequency was found between males (11 out of 965, 1.1%) and females (169 out of 15,362, 1.1%). Besides, five P/LP variants (one in *ATM*, two in *BRCA2*, and two in *PALB2*) were also found in 1117 lung cancer patients (Table S4).

### 3.4 Identification of germline variants with increased risk to lung cancer

As shown in Figure 1 and Table 3, the prevalence of P/LP variants was significantly higher in lung cancer patients (2.24%, 25 out of 1117) than in controls (1.11%, 181 out of 16,327) (*p* < 0.01). Among the 25 P/LP variants identified in the lung cancer patients, four were found in *ATM* (0.36%), six in *BRCA1* (0.54%), seven in *BRCA2* (0.63%), two in *CHEK2* (0.18%), four in *PALB2* (0.36%), and two in *TP53* (0.18%). Meanwhile, of the 181 P/LP variants identified in the controls, 34 variants (0.21%) were found in *ATM*, 21 in *BRCA1* (0.13%), 53 in *BRCA2* (0.32%), 18 in *CHEK2* (0.11%), 32 in *PALB2* (0.20%), and one in *TP53* (0.01%). In addition, P/LP variants were also identified in other genes including *APC*, *CDH1*, *MLH1*, *MSH6*, *PTEN*, and *STK11* in the controls, but not in *MSH2*. P/LP variants in *BRCA2* was the most prevalent in both lung cancer patients and the controls, while only P/LP variants in *BRCA1* and *TP53* genes were found to be significantly associated with lung cancer risk, with odds ratios (ORs) ranging from 4‐fold (*BRCA1*: OR, 4.193; 95%CI, 1.382–10.768) to 29‐fold (*TP53*: OR, 29.281; 95%CI, 1.523–1705.506). Although P/LP variants of *ATM*, *BRCA2*, *CHEK2*, and *PALB2* genes were expected to increase susceptibility with ORs ranging from 1.625 to 1.936, statistical significance was not attained. No P/LP variants were detected in *APC*, *CDH1*, *MLH1*, *MSH2*, *MSH6*, *PTEN*, and *STK11* genes in lung cancer patients, implying these genes might not be associated with lung cancer susceptibility.

In addition to P/LP variants, we found 23 variants previously classified as B/LB, VUS, or unclassified variants in ClinVar database might also be associated with increased risk for lung cancer, with adjusted *p* values <0.05 and ORs varying from 7.322 to infinity (Table 4 and Table S5). These 23 variants were found in exons, introns, or untranslated regions (UTRs) of 11 genes, including *APC*, *ATM*, *BRCA1*, *BRCA2*, *CDH1*, *CHEK2*, *MSH2*, *MSH6*, *PTEN*, *STK11*, and *TP53*. Eight of the 23 variants were nonsynonymous, two were synonymous, while 10 and six were situated in introns and UTRs, respectively. The majority of the 23 variants had no allele frequency information in the 1000 Genome, ExAC, and gnomAD databases, whereas only a few were reported with the maximum allele frequency at 0.1% in East Asian population (Table S5). Our findings indicated P/LP variants of the *BRCA1* and *TP53* genes, as well as 23 rare variants, contributed significantly to lung cancer susceptibility.

**TABLE 4:** Variants associated with increased risk for lung cancer.

Gene	Chr.	Start	End	Ref	Alt.	cDNA change	Location	Type	Prevalence		Odds ratio	95% CI	*p* value	*Adjusted p* value^a^
									Patients	Controls				
*APC*	chr5	1.13E+08	1.13E+08	A	T	c.2378A>T	Exonic	Nonsynonymous	2/1117 (0.18%)	0/16327	Inf	2.75‐Inf	0.004	0.012
*BRCA2*	chr13	32,337,534	32,337,534	G	A	c.3179G>A	Exonic	Nonsynonymous	2/1117 (0.18%)	0/16327	Inf	2.75‐Inf	0.004	0.012
*BRCA2*	chr13	32,354,994	32,354,994	C	T	c.7141C>T	Exonic	Nonsynonymous	2/1117 (0.18%)	0/16327	Inf	2.75‐Inf	0.004	0.012
*CHEK2*	chr22	28,734,685	28,734,685	G	T	c.37C>A	Exonic	Nonsynonymous	2/1117 (0.18%)	0/16327	Inf	2.75‐Inf	0.004	0.012
*BRCA2*	chr13	32,338,204	32,338,204	A	C	c.3849A>C	Exonic	Synonymous	2/1117 (0.18%)	0/16327	Inf	2.75‐Inf	0.004	0.012
*BRCA1*	chr17	43,099,718	43,099,718	A	T	g.43099718A>T	Intronic	–	2/1117 (0.18%)	0/16327	Inf	2.75‐Inf	0.004	0.012
*MSH6*	chr2	47,803,780	47,803,783	AGTC	–	g.47803780_47803783delAGTC	Intronic	–	2/1117 (0.18%)	0/16327	Inf	2.75‐Inf	0.004	0.012
*STK11*	chr19	1,227,890	1,227,890	G	A	c.*314G>A	UTR3	–	2/1117 (0.18%)	0/16327	Inf	2.75‐Inf	0.004	0.012
*ATM*	chr11	1.08E+08	1.08E+08	T	C	c.6765T>C	Exonic	Synonymous	3/1117 (0.27%)	3/16327 (0.02%)	14.646	1.96–109.69	0.005	0.013
*ATM*	chr11	1.08E+08	1.08E+08	G	A	c.7382G>A	Exonic	Nonsynonymous	3/1117 (0.27%)	4/16327 (0.02%)	10.986	1.61–65.08	0.008	0.014
*PTEN*	chr10	87,966,025	87,966,025	C	A	c.*553C>A	UTR3	–	3/1117 (0.27%)	4/16327 (0.02%)	10.986	1.61–65.08	0.008	0.014
*MSH6*	chr2	47,803,386	47,803,386	G	A	g.47803386G>A	Intronic	–	3/1117 (0.27%)	5/16327 (0.03%)	8.789	1.36–45.24	0.011	0.014
*ATM*	chr11	1.08E+08	1.08E+08	A	C	g.108256362A>C	Intronic	–	2/1117 (0.18%)	1/16327 (0.01%)	29.281	1.52–1705.51	0.012	0.014
*ATM*	chr11	1.08E+08	1.08E+08	A	C	g.108256374A>C	Intronic	–	2/1117 (0.18%)	1/16327 (0.01%)	29.281	1.52–1705.51	0.012	0.014
*CDH1*	chr16	68,812,116	68,812,117	TG	–	g.68812116_68812117delTG	Intronic	–	2/1117 (0.18%)	1/16327 (0.01%)	29.281	1.52–1705.51	0.012	0.014
*MSH2*	chr2	47,416,273	47,416,273	C	T	g.47416273C>T	Intronic	–	2/1117 (0.18%)	1/16327 (0.01%)	29.281	1.52–1705.51	0.012	0.014
*MSH6*	chr2	47,801,163	47,801,164	TT	–	g.47801163_47801164delTT	Intronic	–	2/1117 (0.18%)	1/16327 (0.01%)	29.281	1.52–1705.51	0.012	0.014
*PTEN*	chr10	87,969,755	87,969,755	A	G	c.*4283A>G	UTR3	–	2/1117 (0.18%)	1/16327 (0.01%)	29.281	1.52–1705.51	0.012	0.014
*PTEN*	chr10	87,971,766	87,971,766	‐	T	c.*6294‐>T	UTR3	–	2/1117 (0.18%)	1/16327 (0.01%)	29.281	1.52–1705.51	0.012	0.014
*TP53*	chr17	7,669,449	7,669,449	A	G	c.*160T>C	UTR3	–	2/1117 (0.18%)	1/16327 (0.01%)	29.281	1.52–1705.51	0.012	0.014
*MSH6*	chr2	47,803,797	47,803,797	A	C	g.47803797A>C	Intronic	–	3/1117 (0.27%)	6/16327 (0.04%)	7.323	1.18–34.35	0.016	0.018
*BRCA2*	chr13	32,354,916	32,354,916	G	A	c.7063G>A	Exonic	Nonsynonymous	2/1117 (0.18%)	2/16327 (0.01%)	14.633	1.06–201.79	0.023	0.023
*PTEN*	chr10	87,970,155	87,970,155	A	C	c.*4683A>C	UTR3	–	2/1117 (0.18%)	2/16327 (0.01%)	14.633	1.06–201.79	0.023	0.023

^a^ Benjamini‐Hochberg adjusted *p* value.

## 4 DISCUSSION

The potential significance of germline pathogenic variants in lung cancer susceptibility has received a lot of attention in recent years. Through case–control studies carried out in Caucasian, new deleterious germline variants associated with lung cancer susceptibility have been uncovered, which greatly enhanced our understanding of lung cancer heredity.^24^, ^25^, ^26^ However, it has been proposed that the profile of germline variants in Chinese lung cancer patients may differ ethnically from that of western patients.^27^ Case–control studies are needed to uncover ethnic‐specific germline variants that increase the risk of lung cancer in the Chinese population.

Lung cancer, colorectal cancer, and breast cancer were reported to be the first, second, and fourth most frequent malignancies in China in 2020.^28^ P/LP germline variants in *ATM*, *BRCA1*, *BRCA2*, *CDH1*, *CHEK2*, *PALB2*, *PTEN*, *STK11*, and *TP53* genes have been linked to an elevated risk of breast cancer, whereas *APC*, *CHEK2*, *MLH1*, *MSH2*, *MSH6*, *PTEN*, and *STK11* have been identified as causal genes in colorectal cancer.^29^, ^30^ In a recent study, P/LP germline variants in *ATM*, *BRCA1*, *BRCA2*, *CHEK2*, *MSH6*, *PALB2*, and *TP53* were reported to be enriched in lung cancer patients.^27^ Our targeted sequencing panel, which includes the13 genes mentioned above (*APC*, *ATM*, *BRCA1*, *BRCA2*, *CDH1*, *CHEK2*, *MLH1*, *MSH2*, *MSH6*, *PALB2*, *PTEN*, *STK11*, and *TP53*), may be capable of evaluating germline predisposition for lung cancer, colorectal cancer, and breast cancer simultaneously (Figure S1). Using the 13‐genes panel, we attempted to identify germline variants that significantly increase risk to lung cancer in this study. Due to the restricted number of genes in this panel, it is inevitable that other genes that contribute to lung cancer predisposition may be ignored. However, given that there were over 10,000 samples to be investigated in our study, using the 13‐genes panel would significantly reduce the experimental cost compared to a more comprehensive panel.

In this case–control study, we found that germline P/LP variants were considerably more prevalent in lung cancer patients than in the controls (2.24% vs. 1.11%, *p* < 0.01). Our findings demonstrated that germline P/LP variants in the *BRCA1* and *TP53* genes increased the risk of lung cancer in Chinese people. We also discovered 23 rare variants in the *APC*, *ATM*, *BRCA1*, *BRCA2*, *CDH1*, *CHEK2*, *MSH2*, *MSH6*, *PTEN*, *STK11*, and *TP53* genes that may confer increased risk for lung cancer. However, in‐depth mechanistic studies on these rare variants are needed to substantiate their pathogenic roles in lung cancer.

The prevalence of germline *BRCA1* and *BRCA2* P/LP variants was 0.24% and 0.79%, respectively, according to a large survey encompassing 6220 Chinese patients with advanced NSCLC.^11^ Carriers of *BRCA1* and *BRCA2* P/LP variants accounted for 0.79% and 0.60% of 1017 lung cancer patients with adenocarcinoma or squamous cell carcinoma, respectively, in the TCGA cohort.^15^ We found that 1.17% of 1117 unselected Chinese lung cancer patients carried *BRCA1* (0.54%) and *BRCA2* (0.63%) P/LP variants. These findings showed that the proportion of *BRCA1* and *BRCA2* carriers differed between cohorts, with more *BRCA2* carriers discovered in Chinese patients and more *BRCA1* carriers detected in TCGA (non‐Chinese) lung cancer patients. However, this did not imply that *BRCA2* played a more important role in Chinese lung cancer patients than *BRCA1*. In fact, when their prevalence in the controls was taken into account, P/LP variants of *BRCA1* but not *BRCA2* were significantly related with an elevated risk of lung cancer, as demonstrated by our study. Compared to the controls, enrichment of *BRCA1* variants in lung cancer patients was statistically significant, with an OR of 4.193 (95% CI 1.382–10.768, *p* = 0.006), but it was not significant for *BRCA2*. Our findings suggested that, in addition to breast and ovarian cancer, P/LP variants of *BRCA1* also conferred increased risk for lung cancer in Chinese people.

Previous studies revealed that the prevalence of *ATM*, *CHEK2*, *PALB2*, and *TP53* germline P/LP variants ranged from 0.11% to 0.29%, 0.39% to 0.40%, 0.11% to 0.29%, and 0.10% to 0.34%, respectively, in two independent Chinese cohorts of lung cancer patients.^13^, ^14^ However, due to a paucity of data on the prevalence of variants in the controls, it was unclear how much these genes contributed to the risk of lung cancer. Taking the advantage of enrolling large number of controls, we found that despite the fact that P/LP variants of *ATM*, *CHEK2*, *PALB2*, and *TP53* were enriched in lung cancer patients, only *TP53* exhibited statistical significance with an OR of 29.281 (95% CI, 1.523–1705.506, *p* = 0.012). In addition, 23 rare variants in the exonic, intronic, or UTR regions of the *APC*, *ATM*, *BRCA1*, *BRCA2*, *CDH1*, *CHEK2*, *MSH2*, *MSH6*, *PTEN*, *STK11*, and *TP53* genes were also found to be significantly enriched in lung cancer patients, implying that these variants may also predispose risks for lung cancer, which needs to be confirmed in other datasets. Furthermore, while pathogenic germline variants of *MLH1* have been reported in lung cancer patients in Western countries,^31^ we did not discover P/LP variants in the *MLH1* gene in the 1117 Chinese patients with lung cancer, indicating ethnic disparities in CSGs may exist.

Lung cancer diagnosed before the age of 50 was always referred to as early‐onset lung cancer. The clinical and epidemiologic features of early‐onset lung cancer were substantially different from those of elder patients, and genetic factors might play an important role in younger patients.^32^, ^33^, ^34^ Polymorphisms in genes involved in xenobiotic metabolism have been identified as hereditary risk factors for early‐onset lung cancer.^33^ Our results showed that the prevalence of germline P/LP variants in patients with early‐onset lung cancer was significantly higher than in individuals with late‐onset lung cancer (5.9% vs. 1.5%, *p* < 0.01). Similar observation was also reported in a study involving 2984 patients with various types of cancer.^16^ Thus, germline P/LP variants might impose a larger risk in younger patients than in elder ones.

According to a recent study, 1019 out of 2984 patients (34.1%) with various malignancies had a family cancer history in first‐degree relatives, implying that genetic factors might play an important role in multiple types of cancer.^16^ Consistently, 218 out of the 1117 unselected lung cancer patients (19.5%) involved in our study self‐reported a family cancer history in one or more first‐degree relatives. However, P/LP variants were only found in seven of the 218 patients (3.2%), and the majority patients with a family cancer history lacked P/LP variants in the targeted genes we examined. There might be more deleterious variants or risk loci in other genes that contributed to the heritability of lung cancer, and an extended gene panel would improve the possibility of detecting more heritable variants.^16^ However, 18 of the 25 P/LP variant carriers (72%) in our study did not have a family cancer history, which would have been missed if germline testing was offered only on family history‐based criteria. It has been recommended that germline genetic testing be integrated into somatic genomic testing to identify additional clinically actionable variants.^19^, ^35^

This study has several limitations. First, the age and sex of the controls did not match that of the lung cancer patients. The majority of individuals in the control group (94.1%) were female and much younger than lung cancer patients. Consistently, according to a survey carried out in the United States with ~34,000 responders, 94.8% of those who had taken genetic testing were females.^22^ It is likely that young ladies were more willing to undergo routine health checks as well as genetic testing. However, because a germline variant is transferred straight from a parent to a child at conception, age, or sex would not introduce bias into its prevalence. Second, this was a retrospective study. A long‐term follow‐up study for the control group would be of great importance in determining the incidence of malignancies in P/LP variant carriers. Third, the number of CSGs investigated in this study is restricted, and the amplicon‐based NGS approach we applied was incapable of detecting large deletions, duplications, and copy number variations in target genes. Whole exome sequencing could address these issues, but the cost would be much higher when investigating 10,000 samples. Fourth, there was no family cancer history in the controls, and genetic testing was not performed on the family members of P/LP variant carriers, which might limit the generalizability of our findings.

In summary, our results indicated that P/LP variants in the *BRCA1* and *TP53* genes significantly elevated the risk of lung cancer in Chinese people. In addition, we revealed that 23 variants previously classified as B/LB, VUS, or unclassified variants were significantly enriched in lung cancer patients compared to controls. Our findings highlight the need for a case–control study to evaluate the contribution of CSGs to cancer risk.

## AUTHOR CONTRIBUTIONS

**Bing Wei:** Conceptualization (equal); writing – original draft (equal). **Jiadong Zhao:** Data curation (equal). **Jun Li:** Data curation (equal). **Junnan Feng:** Data curation (equal). **Manman Sun:** Data curation (equal). **Zhizhong Wang:** Data curation (equal). **Chao Shi:** Data curation (equal). **Ke Yang:** Data curation (equal). **Yue Qin:** Formal analysis (equal). **Jing Zhang:** Methodology (equal). **Jie Ma:** Conceptualization (equal). **Hui Dong:** Conceptualization (equal); writing – original draft (equal); writing – review and editing (equal).

## FUNDING INFORMATION

This work was supported by funds provided to Dr. Dong by Shanghai General Hospital Start‐up Fund (02.06.01.20.01) and Clinical Research Innovation Plan of Shanghai General Hospital (CTCCR‐2021B03).

## CONFLICT OF INTEREST STATEMENT

The authors declare no conflicts of interest.

## ETHICS STATEMENT

The study was performed in accordance with the Declaration of Helsinki and approved by the Ethics Committee of Henan Cancer Hospital and Shanghai Ethics Committee for Clinical Research.

## CONSENT TO PARTICIPATE

Written informed consent was obtained from all subjects.

## Supporting information


Figure S1

Table S1

Table S2

Table S3

Table S4

Table S5

## Data Availability

The data that support the findings of this study are available from the corresponding author upon reasonable request.

## References

[1] Sung H , Ferlay J , Siegel RL , et al. Global cancer statistics 2020: GLOBOCAN estimates of incidence and mortality worldwide for 36 cancers in 185 countries. CA Cancer J Clin. 2021;71(3):209‐249. doi:10.3322/caac.21660 33538338

[2] Loeb LA , Ernster VL , Warner KE , Abbotts J , Laszlo J . Smoking and lung cancer: an overview. Cancer Res. 1984;44(12 Pt 1):5940‐5958. 6388830

[3] Barone‐Adesi F , Chapman RS , Silverman DT , et al. Risk of lung cancer associated with domestic use of coal in Xuanwei, China: retrospective cohort study. BMJ. 2012;345:e5414. doi:10.1136/bmj.e5414 22936785 PMC3431444

[4] Mollo F , Magnani C , Bo P , Burlo P , Cravello M . The attribution of lung cancers to asbestos exposure: a pathologic study of 924 unselected cases. Am J Clin Pathol. 2002;117(1):90‐95. doi:10.1309/DEDU-V6UC-587A-9CGD 11789737

[5] Mucci LA , Hjelmborg JB , Harris JR , et al. Nordic twin study of cancer (NorTwinCan) collaboration. Familial risk and heritability of cancer among twins in Nordic countries. JAMA. 2016;315(1):68‐76. doi:10.1001/jama.2015.17703 26746459 PMC5498110

[6] Bossé Y , Amos CI . A decade of GWAS results in lung cancer. Cancer Epidemiol Biomarkers Prev. 2018;27(4):363‐379. doi:10.1158/1055-9965 28615365 PMC6464125

[7] Dai J , Lv J , Zhu M , et al. Identification of risk loci and a polygenic risk score for lung cancer: a large‐scale prospective cohort study in Chinese populations. Lancet Respir Med. 2019;7(10):881‐891. doi:10.1016/S2213-2600(19)30144-4 31326317 PMC7015703

[8] Yu HA , Arcila ME , Harlan Fleischut M , et al. Germline EGFR T790M mutation found in multiple members of a familial cohort. J Thorac Oncol. 2014;9(4):554‐558. doi:10.1097/JTO.0000000000000052 24736080 PMC4412273

[9] Xiong D , Wang Y , Kupert E , et al. A recurrent mutation in PARK2 is associated with familial lung cancer. Am J Hum Genet. 2015;96(2):301‐308. doi:10.1016/j.ajhg.2014.12.016 25640678 PMC4320264

[10] Chen HY , Yu SL , Ho BC , et al. R331W missense mutation of oncogene YAP1 is a germline risk allele for lung adenocarcinoma with medical Actionability. J Clin Oncol. 2015;33(20):2303‐2310. doi:10.1200/JCO.2014.59.3590 26056182

[11] Hu X , Yang D , Li Y , et al. Prevalence and clinical significance of pathogenic germline BRCA1/2 mutations in Chinese non‐small cell lung cancer patients. Cancer Biol Med. 2019;16(3):556‐564. doi:10.20892/j.issn.2095-3941.2018.0506 31565484 PMC6743617

[12] Lu S , Yu Y , Li Z , et al. EGFR and ERBB2 germline mutations in Chinese lung cancer patients and their roles in genetic susceptibility to cancer. J Thorac Oncol. 2019;14(4):732‐736. doi:10.1016/j.jtho.2018.12.006 30610926

[13] Tian P , Cheng X , Zhao Z , et al. Spectrum of pathogenic germline mutations in Chinese lung cancer patients through next‐generation sequencing. Pathol Oncol Res. 2020;26(1):109‐114. doi:10.1007/s12253-019-00771-5 31721094

[14] Liu M , Liu X , Suo P , et al. The contribution of hereditary cancer‐related germline mutations to lung cancer susceptibility. Transl Lung Cancer Res. 2020;9(3):646‐658. doi:10.21037/tlcr-19-403 32676327 PMC7354149

[15] Huang KL , Mashl RJ , Wu Y , et al. Pathogenic germline variants in 10,389 adult cancers. Cell. 2018;173(2):355‐370.e14. doi:10.1016/j.cell.2018.03.039 29625052 PMC5949147

[16] Samadder NJ , Riegert‐Johnson D , Boardman L , et al. Comparison of universal genetic testing vs guideline‐directed targeted testing for patients with hereditary cancer syndrome. JAMA Oncol. 2021;7(2):230‐237. doi:10.1001/jamaoncol.2020.6252 33126242 PMC7600058

[17] Dong H , Chandratre K , Qin Y , et al. Prevalence of BRCA1/BRCA2 pathogenic variation in Chinese Han population. J Med Genet. 2021;58(8):565‐569. doi:10.1136/jmedgenet-2020-106970 32467295

[18] Richards S , Aziz N , Bale S , et al. Standards and guidelines for the interpretation of sequence variants: a joint consensus recommendation of the American College of Medical Genetics and Genomics and the Association for Molecular Pathology. Genet Med. 2015;17(5):405‐424. doi:10.1038/gim.2015.30 25741868 PMC4544753

[19] Noguchi R , Shen J . Factors affecting participation in health checkups: evidence from Japanese survey data. Health Policy. 2019;123(4):360‐366. doi:10.1016/j.healthpol.2018.10.013 30691696

[20] Rotarou ES , Sakellariou D . Determinants of utilisation rates of preventive health services: evidence from Chile. BMC Public Health. 2018;18(1):839. doi:10.1186/s12889-018-5763-4 29976166 PMC6034328

[21] d'Agincourt‐Canning L , Baird P . Genetic testing for hereditary cancers: the impact of gender on interest, uptake and ethical considerations. Crit Rev Oncol Hematol. 2006;58(2):114‐123. doi:10.1016/j.critrevonc.2006.03.001 16600617

[22] Pritchard CC . New name for breast‐cancer syndrome could help to save lives. Nature. 2019;571(7763):27‐29. doi:10.1038/d41586-019-02015-7 31270479

[23] Radice P , De Summa S , Caleca L , Tommasi S . Unclassified variants in BRCA genes: guidelines for interpretation. Ann Oncol. 2011;22(Suppl 1):i18‐i23. doi:10.1093/annonc/mdq661 21285146

[24] Liu Y , Lusk CM , Cho MH , et al. Rare variants in known susceptibility loci and their contribution to risk of lung cancer. J Thorac Oncol. 2018;13(10):1483‐1495. doi:10.1016/j.jtho.2018.06.016 29981437 PMC6366341

[25] Liu Y , Xia J , McKay J , et al. Rare deleterious germline variants and risk of lung cancer. NPJ Precis Oncol. 2021;5(1):12. doi:10.1038/s41698-021-00146-7 33594163 PMC7887261

[26] Sang J , Zhang T , Kim J , et al. Rare germline deleterious variants increase susceptibility for lung cancer. Hum Mol Genet. 2022;31(20):3558‐3565. doi:10.1093/hmg/ddac123 35717579 PMC9558843

[27] Peng W , Li B , Li J , et al. Clinical and genomic features of Chinese lung cancer patients with germline mutations. Nat Commun. 2022;13(1):1268. doi:10.1038/s41467-022-28840-5 35273153 PMC8913621

[28] Cao W , Chen HD , Yu YW , Li N , Chen WQ . Changing profiles of cancer burden worldwide and in China: a secondary analysis of the global cancer statistics 2020. Chin Med J (Engl). 2021;134(7):783‐791. doi:10.1097/CM9.0000000000001474 33734139 PMC8104205

[29] Easton DF , Pharoah PD , Antoniou AC , et al. Gene‐panel sequencing and the prediction of breast‐cancer risk. N Engl J Med. 2015;372(23):2243‐2257. doi:10.1056/NEJMsr1501341 26014596 PMC4610139

[30] Valle L , Vilar E , Tavtigian SV , Stoffel EM . Genetic predisposition to colorectal cancer: syndromes, genes, classification of genetic variants and implications for precision medicine. J Pathol. 2019;247(5):574‐588. doi:10.1002/path.5229 30584801 PMC6747691

[31] Lincoln SE , Nussbaum RL , Kurian AW , et al. Yield and utility of germline testing following tumor sequencing in patients with cancer. JAMA Netw Open. 2020;3(10):e2019452. doi:10.1001/jamanetworkopen.2020.19452 33026450 PMC7542302

[32] Etzel CJ , Lu M , Merriman K , Liu M , Vaporciyan A , Spitz MR . An epidemiologic study of early onset lung cancer. Lung Cancer. 2006;52(2):129‐134. doi:10.1016/j.lungcan.2005.11.018 16564601

[33] Timofeeva M , Kropp S , Sauter W , et al. Genetic polymorphisms of MPO, GSTT1, GSTM1, GSTP1, EPHX1 and NQO1 as risk factors of early‐onset lung cancer. Int J Cancer. 2010;127(7):1547‐1561. doi:10.1002/ijc.25175 20091863

[34] Li X , Hemminki K . Inherited predisposition to early onset lung cancer according to histological type. Int J Cancer. 2004;112(3):451‐457. doi:10.1002/ijc.20436 15382071

[35] Robson ME , Bradbury AR , Arun B , et al. American Society of Clinical Oncology policy statement update: genetic and genomic testing for cancer susceptibility. J Clin Oncol. 2015;33(31):3660‐3667. doi:10.1200/JCO.2015.63.0996 26324357

