# Supplementary figures and images for: Pathogenic germline variants in BRCA1 and TP53 increase lung cancer risk in Chinese
Source: Cancer Med. 2023 Nov 6;12(23):21219–28. doi: 10.1002/cam4.6692 (PMC10726856; doi:10.1002/cam4.6692)

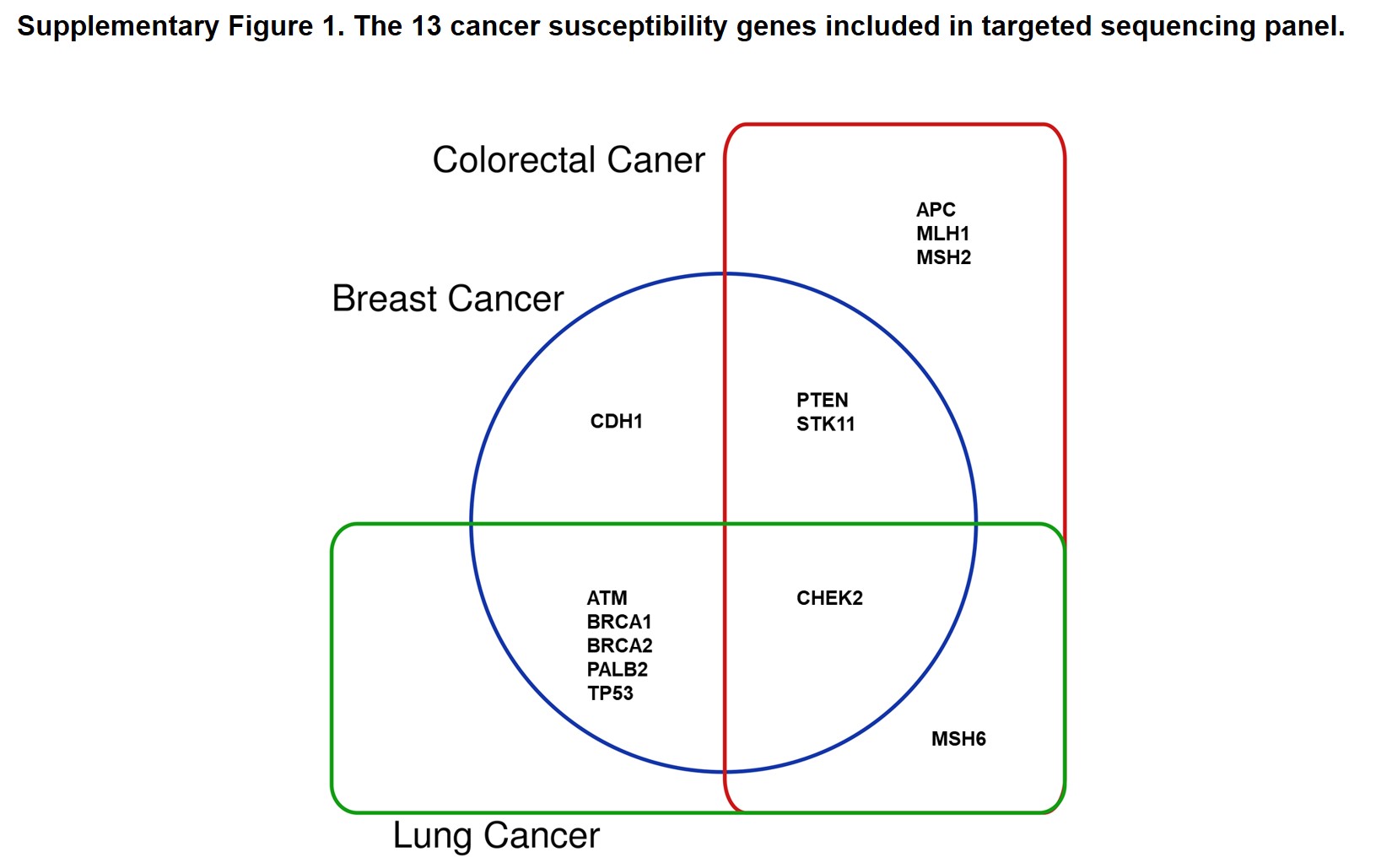

Supplement: Supplementary file 1 — Figure S1 [file CAM4-12-21219-s005.jpg]
